# Occupational Sitting Time, Leisure Physical Activity, and All-Cause and Cardiovascular Disease Mortality

**DOI:** 10.1001/jamanetworkopen.2023.50680

**Published:** 2024-01-19

**Authors:** Wayne Gao, Mattia Sanna, Yea-Hung Chen, Min-Kuang Tsai, Chi-Pang Wen

**Affiliations:** 1 PhD Program in Global Health and Health Security, College of Public Health, Taipei Medical University, Taipei City, Taiwan; 2 Master’s Program in Global Health and Health Security, College of Public Health, Taipei Medical University, Taipei City, Taiwan; 3 Department of Epidemiology and Biostatistics, University of California, San Francisco; 4 Division of Nephrology, Department of Internal Medicine, Shuang Ho Hospital, Taipei Medical University, New Taipei City, Taiwan; 5 Institute of Population Health Science, National Health Research Institutes, Miaoli County, Taiwan; 6 China Medical University Hospital, Taichung City, Taiwan

## Abstract

**Importance:**

For the first time, the 2020 World Health Organization guidelines on physical activity recommended reducing sedentary behaviors owing to their health consequences. Less is known on the specific association of prolonged occupational sitting with health, especially in the context of low physical activity engagement.

**Objective:**

To quantify health risks associated with prolonged occupational sitting and to determine whether there is a certain threshold of physical activity that may attenuate it.

**Design, Setting, and Participants:**

This prospective cohort study included participants in a health surveillance program in Taiwan who were followed-up between 1996 and 2017. Data on occupational sitting, leisure-time physical activity (LTPA) habits, lifestyle, and metabolic parameters were collected. Data analysis was performed in December 2020.

**Main Outcomes and Measures:**

The all-cause and cardiovascular disease (CVD) mortality associated with 3 occupational sitting volumes (mostly sitting, alternating sitting and nonsitting, and mostly nonsitting) were analyzed applying multivariable Cox regression models to calculate the hazard ratios (HRs) for all participants and by subgroups, including 5 LTPA levels and a personal activity intelligence (PAI)–oriented metric. Deaths occurring within the initial 2 years of follow-up were excluded to prevent reverse causality.

**Results:**

The total cohort included 481 688 participants (mean [SD] age, 39.3 [12.8] years; 256 077 women [53.2%]). The study recorded 26 257 deaths during a mean (SD) follow-up period of 12.85 (5.67) years. After adjusting for sex, age, education, smoking, drinking, and body mass index, individuals who mostly sat at work had a 16% higher all-cause mortality risk (HR, 1.16; 95% CI, 1.11-1.20) and a 34% increased mortality risk from CVD (HR, 1.34; 95% CI, 1.22-1.46) compared with those who were mostly nonsitting at work. Individuals alternating sitting and nonsitting at work did not experience increased risk of all-cause mortality compared with individuals mostly nonsitting at work (HR, 1.01; 95% CI, 0.97-1.05). For individuals mostly sitting at work and engaging in low (15-29 minutes per day) or no (<15 minutes per day) LTPA, an increase in LTPA by 15 and 30 minutes per day, respectively, was associated with a reduction in mortality to a level similar to that of inactive individuals who mostly do not sit at work. In addition, individuals with a PAI score exceeding 100 experienced a notable reduction in the elevated mortality risk associated with prolonged occupational sitting.

**Conclusions and Relevance:**

As part of modern lifestyles, prolonged occupational sitting is considered normal and has not received due attention, even though its deleterious effect on health outcomes has been demonstrated. In this study, alternating between sitting and nonsitting at work, as well as an extra 15 to 30 minutes per day of LTPA or achieving a PAI score greater than 100, attenuated the harms of prolonged occupational sitting. Emphasizing the associated harms and suggesting workplace system changes may help society to denormalize this common behavior, similar to the process of denormalizing smoking.

## Key Points

**Question:**

What are the health outcomes associated with prolonged occupational sitting in the context of various levels of physical activity among apparently healthy individuals?

**Findings:**

In this cohort study involving 481 688 individuals over a mean follow-up period of 12.85 years, individuals who predominantly engaged in sitting at work exhibited a higher risk of mortality from all causes (16%) and cardiovascular disease (34%) compared with those who predominantly did not sit, even after adjusting for sex, age, education, smoking, drinking, and body mass index. Individuals who predominantly sit at work would need to engage in an additional 15 to 30 minutes of physical activity per day to mitigate this increased risk and reach the same level of risk as individuals who predominantly do not sit at work.

**Meaning:**

These findings suggest that reducing prolonged sitting in the workplace and/or increasing the volume or intensity of daily physical activity may be beneficial in mitigating the elevated risks of all-cause mortality and cardiovascular disease associated with prolonged occupational sitting.

## Teaser

This cohort study examines the health outcomes associated with prolonged occupational sitting and assesses the additional amount of physical activity that may be needed to attenuate it.

## Introduction

Modern lifestyles have become increasingly sedentary,^1^ with prolonged sitting now pervasive as an integral part of normal life,^2^ despite the fact that, with some exceptions,^3^ the scientific literature agrees on its deleterious effects. For the first time in 2020, the World Health Organization guidelines on physical activity recommended reducing sedentary behaviors because of their health consequences,^4^ which accords with similar recent physical activities guidelines published in the US in 2018^5^ and in the UK in 2019^6^ that also discourage prolonged sitting. However, adhering to these recommendations, particularly in workplace settings, remains challenging and is not well supported.

Few studies have specifically examined prolonged occupational sitting. However, several studies have found increased mortality among prolonged sitters from all causes,^7,8,9,10,11^ as well as from cardiovascular disease (CVD),^12^ cancer,^13,14^ and diabetes.^15,16^ For example, a cohort study^8^ of more than 200 000 individuals in Australia found a dose-response association between prolonged sitting and all-cause mortality. However, associations between occupational prolonged sitting and health outcomes are not consistently demonstrated. Prospective studies have generally found that occupational prolonged sitting increases the risk of diabetes, for example, but not cancer.^17^ Similarly, an analysis found that associations between prolonged sitting and health outcomes are dependent on sex.^18^ In addition, the health risks of prolonged sitting have been shown to be independent of leisure-time physical inactivity.^19,20^ Working long hours shares similarities with prolonged sitting in terms of health impacts, demonstrating dose-response associations with coronary heart disease and stroke.^21,22^ Thus, one would expect similar, if not worse health consequences for prolonged occupational sitting.

Another important issue is the level of leisure-time physical activity (LTPA) needed to attenuate the health repercussions of prolonged occupational sitting. A meta-analysis by Ekelund and colleagues^20^ concluded that the risk of prolonged sitting could be eliminated with 60 to 75 minutes per day of exercise, approximately 4 to 5 times higher than the current World Health Organization recommendation.^23^ Later, using objective accelerometer-measured physical activity in a harmonized meta-analysis, Ekelund and colleagues^24^ concluded that approximately 30 to 40 minutes of moderate-to-vigorous intensity physical activity per day attenuates the prolonged sitting mortality risk. Meeting the current physical activity guidelines seems to effectively eliminate all-cause and CVD mortality risk associated with sitting among the least physical active adults.^24^ However, it is less clear whether the findings apply to occupational prolonged sitting. In this context, Zisko and colleagues^25^ suggest that the adverse health consequences of prolonged sedentary behavior may be mitigated by maintaining a specified weekly threshold of physical activity, as assessed through the personal activity intelligence (PAI) metric. PAI is a novel metric for tracking physical activity, unique for its ability to incorporate personalized heart rate in response to activity, irrespective of the specific nature of the physical activity undertaken.^25,26^

Our study used a specific approach, comparing nonsitters at a specific LTPA level (used as the reference) with sitters at the same or higher LTPA levels. This comparison helps to identify the LTPA level at which no significant difference emerges compared with the nonsitting reference, a pivotal point in ensuring clear and accurate interpretation of the results. In this study, we aim to (1) assess the health risks of prolonged sitting at work on all-cause and CVD mortality, and (2) quantify the requisite amount of LTPA and intensity, as measured by PAI, needed to mitigate these risks for individuals mostly sitting at work at a level comparable to those mostly nonsitting at work.

## Methods

### Study Design, Population, and Data Collection

Our cohort included individuals aged 20 years and older who participated in a membership-based annual to biannual health checkup program in Taiwan, between 1996 and 2017 (eFigure 1 in Supplement 1). These apparently healthy participants were followed-up for a mean (SD) period of 12.85 (5.67) years, which equates to 6 186 949 person-years at follow-up.

We selected only participants without preexisting CVD diagnoses at baseline. During each visit, participants completed a questionnaire on medical history and lifestyle risk factors, with biological test specimens collected for testing. Previous studies^27,28^ provide comprehensive details on the entire program and data collection methods. Participants provided written informed consent through the questionnaires. This cohort study was approved by the Research Ethics Committee at China Medical University, Taiwan. This study follows the Strengthening the Reporting of Observational Studies in Epidemiology (STROBE) reporting guidelines for cohort studies.

### Measurement of Prolonged Occupational Sitting, Physical Activity, and Adverse Health Outcomes

Occupational sitting status was categorized into 3 groups (mostly sitting, alternating sitting and nonsitting, and mostly nonsitting) according to the answer to the following question included in the questionnaire: “What is your level of physical activity at work?” The response options included mostly sitting, mostly sitting and standing while performing repetitive motions in the course of work, and standing and walking around most of the time.

The LTPA status was determined through 2 multiple-choice questions. The first asked the participants to classify all their weekly LTPAs in the last month into 4 intensity categories: light (eg, walking), moderate (eg, brisk walking), medium vigorous (eg, jogging), or highly vigorous (eg, running). The second asked about the weekly duration of each LTPA in the last month. Further details can be found in Wen et al.^27^ On the basis of their answers, participants were classified into 5 LTPA groups by metabolic equivalent of task (MET) hours per week: inactive (<3.75 MET hours per week or <15 minutes per day), low (ie, engaged in low LTPA; 3.75-7.49 MET hours per week or 15-29 minutes per day), medium (7.50-16.49 MET hours per week or 30-59 minutes per day), high (16.5-25.49 MET hours per week or 60-89 minutes per day), and very high (≥25.5 MET hours per week or ≥90 minutes per day). The codification proposed by Ainsworth and colleagues^29^ was adopted.

In addition, we estimated participants’ physical activity, using the PAI, which converted self-reported activity level into relative intensities, approximating 44%, 73%, and 83% of heart rate reserve values to correspond to low, moderate, and vigorous intensity physical activities, respectively. The individual PAI scores were then derived by combining exercise volume with the intensity calculations based on heart rate reserve.^26,30^

We used baseline measures of prolonged occupational sitting, LTPA, and PAI since follow-up survey responses were not available for 59% of study participants. Accordingly, all the covariates in this study, including age, sex, education, smoking status, drinking status, body mass index (BMI; calculated as weight in kilograms divided by height in meters squared), systolic blood pressure, fasting blood sugar level, and total cholesterol, were assessed at baseline.

Nonetheless, because cohort participants completed the questionnaire multiple times during follow-up, we validated the questionnaire for LTPA by examining the reliability and consistency of answers from 2 visits. The answers from the 2 visits were considerably consistent, as exemplified by a Spearman rank correlation coefficient of 0.61 (*P* < .001) for occupational prolonged sitting, measured using data from 2 different visits (mean [SD] interval, 2.20 [2.06] years) (eTable 1 in Supplement 1). Details of validation of the cohort questionnaire are reported in a previous publication.^27^

Deaths were ascertained by linking each participant’s identification number to the Taiwan National Death Registry, excluding the first 2 years of the follow-up period to avoid reverse causality (ie, sicknesses or poor health causing a sedentary lifestyle). Our analysis encompassed all-cause mortality; we focused on *International Classification of Diseases, Ninth Revision *(*ICD-9*) codes 001 to 998 before 2008 and *International Statistical Classification of Diseases and Related Health Problems, Tenth Revision *(*ICD-10*) codes A00 to Y98 after 2009. For CVD mortality, we used *ICD-9* codes 390 to 459 before 2008 and *ICD-10* codes I00 to I99 after 2009.

### Statistical Analysis

Data analysis was performed in December 2020. First, we fit Cox proportional hazards models to obtain hazard ratios (HRs) for all-cause and CVD mortality relative to a reference of mostly nonsitting. Then, we repeated this analysis stratifying by subgroups, including sex, individuals younger than 60 years, individuals aged 60 years and older, smoking status, BMI 25 or higher, BMI 30 or higher, individuals with hypertension, and individuals with diabetes. We also conducted sensitivity analyses on the so-called expanded CVD mortality, consisting of cardiovascular disease (*ICD-9 *codes 390-459), plus diabetes (*ICD-9* code 250), plus kidney diseases (*ICD-9* codes 580-589), to account for the common practice of physicians in Taiwan to enter diabetes as the underlying cause of death.^31,32,33,34^ In addition, we conducted an analysis excluding individuals with existing health conditions, such as hypertension, high BMI, and diabetes, at baseline as a sensitivity analysis to confirm the primary results.

Finally, we fit a Cox model between occupational sitting status and LTPA to obtain HRs of sitting-LTPA groups compared with the reference of mostly sitting and no LTPA. We also used the model to identify the amount of exercise needed to offset the risk from sitting. The models were adjusted for sex, age, education, smoking, drinking, and BMI. A similar analysis focused on all-cause mortality was conducted with PAI. The observations were right censored. The date of study entry was the date of joining the cohort (1996 the earliest), while the time of exit was December 31, 2017, or the date of death if earlier. The proportional hazards assumption was assessed by testing the interaction between occupational prolonged sitting and time (year). In detail, the correlation between Schoenfeld residuals and time was examined, and no evidence of violation of the assumption was detected because the interaction was not significant. Two-sided *P* < .05 was considered statistically significant. The data analysis was conducted at the Health and Welfare Data Science Center using SAS statistical software version 9.4 (SAS Institute).

## Results

The total cohort included 481 688 participants (mean [SD] age, 39.3 [12.8] years; 256 077 women [53.2%]). A majority of participants (290 075 participants [60.2%]) were in the mostly sitting group, whereas 51 403 (10.7%) were in the nonsitting group and 140 210 (29.1%) were in the alternating sitting and nonsitting group (Table 1). Physical activity varied across sitting groups, with 47.5% of the mostly sitting group (135 378 participants) describing themselves as physically inactive, compared with 51.7% (71 025 participants) in the alternating sitting and nonsitting group, and 57.2% (28 618 participants) in the mostly nonsitting group. Compared with people in the alternating and mostly nonsitting group, people in the mostly sitting group tended to be younger and more educated (college or higher, 141 500 participants [49.4%] in the sitting group vs 32 236 participants [23.3%] in the alternating sitting and nonsitting group and 9070 participants [18.0%] in the mostly nonsitting group), had fewer lifestyle risks like smoking (50 019 participants [17.7%] in the sitting group vs 33 755 participants [25.0%] in the alternating group and 16 817 participants [33.9%] in the nonsitting group) or drinking (17 625 participants [6.3%] in the sitting group vs 12 287 participants [9.2%] in the alternating group and 7168 participants [14.8%] in the nonsitting group), and were less likely to be overweight or obese (BMI ≥25, 74 143 participants [25.6%] in the sitting group vs 39 407 participants [28.1%] and 15 529 participants [30.2%]) or to have hypertension (systolic blood pressure ≥140 mm Hg, 47 420 participants [16.3%] in the sitting group vs 25 414 participants [18.1%] in the alternating group and 10 511 participants [20.4%] in the nonsitting group) (Table 1). Among the participants selected for the study, only a few did not complete all sections of the questionnaire, resulting in 1.4% missing data for education level, 2.9% for smoking status, 4.4% for drinking status, and 2.0% for physical activity level. Data were more than 99% complete for biological tests, including those for serum creatinine, glomerular filtration rate, urine protein test, blood pressure, BMI, and blood glucose. Therefore, prevalence and risks of all-cause and CVD mortality in this study were minimally affected by the missing data. A flow diagram includes excluding and missing data is included in eFigure 2 in Supplement 1.

**Table 1.** Distribution of Sociodemographic Characteristics, Smoking and Drinking, Leisure-Time Physical Activity, and Metabolic Indicators by Occupational Sitting Status

Characteristic	Participants, No. (%)			
	Total	Mostly sitting	Alternating sitting and nonsitting	Mostly nonsitting
Age, y				
20-39	286 867 (59.6)	178 913 (61.7)	82 060 (58.5)	25 894 (50.4)
40-59	147 257 (30.6)	83 620 (28.8)	44 482 (31.7)	19 155 (37.3)
≥60	47 564 (9.9)	27 542 (9.5)	13 668 (9.7)	6354 (12.4)
Sex				
Male	225 611 (46.8)	128 404 (44.3)	64 141 (45.7)	33 066 (64.3)
Female	256 077 (53.2)	161 671 (55.7)	76 069 (54.3)	18 337 (35.7)
Education				
Middle school or lower	93 260 (19.6)	37 911 (13.2)	36 038 (26.1)	19 311 (38.3)
High school	99 827 (21.0)	46 309 (16.2)	39 500 (28.6)	14 018 (27.8)
Junior college	99 166 (20.9)	60 726 (21.2)	30 435 (22.0)	8005 (15.9)
College or higher	182 806 (38.5)	141 500 (49.4)	32 236 (23.3)	9070 (18.0)
Smoking status				
Never smoker	338 745 (72.4)	216 833 (76.6)	93 362 (69.1)	28 550 (57.6)
Former smoker	28 306 (6.1)	16 071 (5.7)	8038 (5.9)	4197 (8.5)
Current smoker	100 611 (21.5)	50 019 (17.7)	33 775 (25.0)	16 817 (33.9)
Drinking status				
Nondrinker	373 541 (81.2)	235 079 (84.2)	104 659 (78.8)	33 803 (69.9)
Moderate drinker	49 679 (10.8)	26 338 (9.4)	15 934 (12.0)	7407 (15.3)
Regular drinker	37 080 (8.1)	17 625 (6.3)	12 287 (9.2)	7168 (14.8)
Leisure-time physical activity				
Inactive	235 021 (49.8)	135 378 (47.5)	71 025 (51.7)	28 618 (57.2)
Low	125 734 (26.6)	81 556 (28.6)	34 367 (25.0)	9811 (19.6)
Medium	70 196 (14.9)	44 312 (15.6)	19 787 (14.4)	6097 (12.2)
High	26 120 (5.5)	15 552 (5.5)	7643 (5.6)	2925 (5.8)
Very high	15 000 (3.2)	7965 (2.8)	4433 (3.2)	2602 (5.2)
Body mass index^a^				
<18.5	43 080 (8.9)	27 930 (9.6)	11 659 (8.3)	3491 (6.8)
18.5-24.9	309 346 (64.2)	187 863 (64.8)	89 111 (63.6)	32 372 (63.0)
25-29.9	108 616 (22.6)	62 252 (21.5)	33 214 (23.7)	13 150 (25.6)
≥30	20 463 (4.2)	11 891 (4.1)	6193 (4.4)	2379 (4.6)
Systolic blood pressure, mm Hg				
<140	398 343 (82.7)	242 655 (83.7)	114 796 (81.9)	40 892 (79.6)
≥140 or taking medication	83 345 (17.3)	47 420 (16.3)	25 414 (18.1)	10 511 (20.4)
Fasting blood glucose level, mg/dL				
<126	458 481 (95.2)	276 273 (95.2)	133 544 (95.2)	48 664 (94.7)
≥126 or taking medication	23 207 (4.8)	13 802 (4.8)	6666 (4.8)	2739 (5.3)
Total cholesterol, mg/dL				
<240	426 931 (88.7)	257 678 (88.9)	124 016 (88.5)	45 237 (88.1)
≥240 or taking medication	54 487 (11.3)	32 258 (11.1)	16 114 (11.5)	6115 (11.9)

SI conversion factors: To convert cholesterol to millimoles per liter, multiply by 0.0259; glucose to millimoles per liter, multiply by 0.0555.

^a^ Body mass index is calculated as weight in kilograms divided by height in meters squared.

During the entire follow-up period of 12.85 years, the study recorded 26 257 deaths, 15 045 (57.3%) occurring in individuals mostly sitting at work (Table 2). There were 5371 CVD-related deaths, with 3234 (60.2%) of these occurring in the mostly sitting group. After adjusting for sex, age, education, smoking, drinking, and BMI, individuals mostly sitting at work had a 16% higher risk of dying by all causes (HR, 1.16; 95% CI, 1.11-1.20) and 34% higher risk of dying from CVD (HR, 1.34; 95% CI, 1.22-1.46), compared with the mostly nonsitting group. Individuals alternating sitting and nonsitting at work did not experience increased risk for all-cause mortality compared with individuals mostly nonsitting at work (HR, 1.01; 95% CI, 0.97-1.05).

**Table 2.** Overall and Subgroup Cox Regression Analysis Output

Population	All-cause		Cardiovascular disease	
	Deaths, No.	HR (95% CI)	Deaths, No.	HR (95% CI)
All participants				
Mostly sitting	15 045	1.16 (1.11-1.20)	3234	1.34 (1.22-1.46)
Alternating sitting and nonsitting	7257	1.01 (0.97-1.05)	1407	1.11 (1.00-1.22)
Mostly nonsitting	3955	1 [Reference]	730	1 [Reference]
Men				
Mostly sitting	8077	1.13 (1.08-1.19)	1784	1.32 (1.19-1.47)
Alternating sitting and nonsitting	3658	1.01 (0.96-1.06)	703	1.09 (0.97-1.22)
Mostly nonsitting	3084	1 [Reference]	567	1 [Reference]
Women				
Mostly sitting	6968	1.21 (1.12-1.31)	1450	1.29 (1.07-1.55)
Alternating sitting and nonsitting	3599	1.03 (0.95-1.12)	704	1.09 (0.90-1.31)
Mostly nonsitting	871	1 [Reference]	163	1 [Reference]
Age <60 y				
Mostly sitting	5563	1.06 (1.00-1.12)	925	1.33 (1.14-1.54)
Alternating sitting and nonsitting	3665	0.99 (0.93-1.05)	578	1.18 (1.01-1.38)
Mostly nonsitting	1962	1 [Reference]	305	1 [Reference]
Age ≥60 y				
Mostly sitting	9482	1.23 (1.16-1.29)	2309	1.32 (1.17-1.48)
Alternating sitting and nonsitting	3592	1.02 (0.96-1.08)	829	1.06 (0.93-1.20)
Mostly nonsitting	1993	1 [Reference]	425	1 [Reference]
Smokers				
Mostly sitting	5561	1.14 (1.08-1.20)	1199	1.27 (1.12-1.43)
Alternating sitting and nonsitting	2671	1.01 (0.95-1.07)	497	1.05 (0.92-1.20)
Mostly nonsitting	2190	1 [Reference]	398	1 [Reference]
Never smokers				
Mostly sitting	8737	1.17 (1.11-1.24)	1875	1.40 (1.23-1.61)
Alternating sitting and nonsitting	4180	1.02 (0.96-1.08)	836	1.17 (1.01-1.35)
Mostly nonsitting	1560	1 [Reference]	281	1 [Reference]
Body mass index ≥25^a^				
Mostly sitting	5835	1.13 (1.06-1.21)	1433	1.42 (1.23-1.65)
Alternating sitting and nonsitting	2805	0.98 (0.91-1.05)	605	1.15 (0.98-1.34)
Mostly nonsitting	1350	1 [Reference]	271	1 [Reference]
Body mass index ≥30^a^				
Mostly sitting	995	1.20 (1.01-1.42)	265	1.72 (1.17-2.54)
Alternating sitting and nonsitting	436	0.96 (0.80-1.16)	103	1.31 (0.87-1.98)
Mostly nonsitting	184	1 [Reference]	39	1 [Reference]
With hypertension				
Mostly sitting	6627	1.08 (1.02-1.14)	954	1.27 (1.09-1.47)
Alternating sitting and nonsitting	3658	0.96 (0.91-1.02)	471	1.06 (0.90-1.25)
Mostly nonsitting	2206	1 [Reference]	295	1 [Reference]
Without hypertension				
Mostly sitting	8418	1.21 (1.14-1.28)	2280	1.28 (1.03-1.58)
Alternating sitting and nonsitting	3599	1.01 (0.95-1.08)	936	0.96 (0.76-1.22)
Mostly nonsitting	1749	1 [Reference]	435	1 [Reference]
Without diabetes				
Mostly sitting	11 514	1.12 (1.07-1.17)	2449	1.33 (1.20-1.47)
Alternating sitting and nonsitting	5959	1.00 (0.95-1.04)	1158	1.13 (1.01-1.26)
Mostly nonsitting	3347	1 [Reference]	610	1 [Reference]
With diabetes				
Mostly sitting	3531	1.20 (1.09-1.32)	785	1.28 (1.03-1.58)
Alternating sitting and nonsitting	1298	0.97 (0.88-1.08)	249	0.96 (0.76-1.22)
Mostly nonsitting	608	1 [Reference]	120	1 [Reference]

Abbreviation: HR, hazard ratio.

^a^ Body mass index is calculated as weight in kilograms divided by height in meters squared.

**Figure 1.**
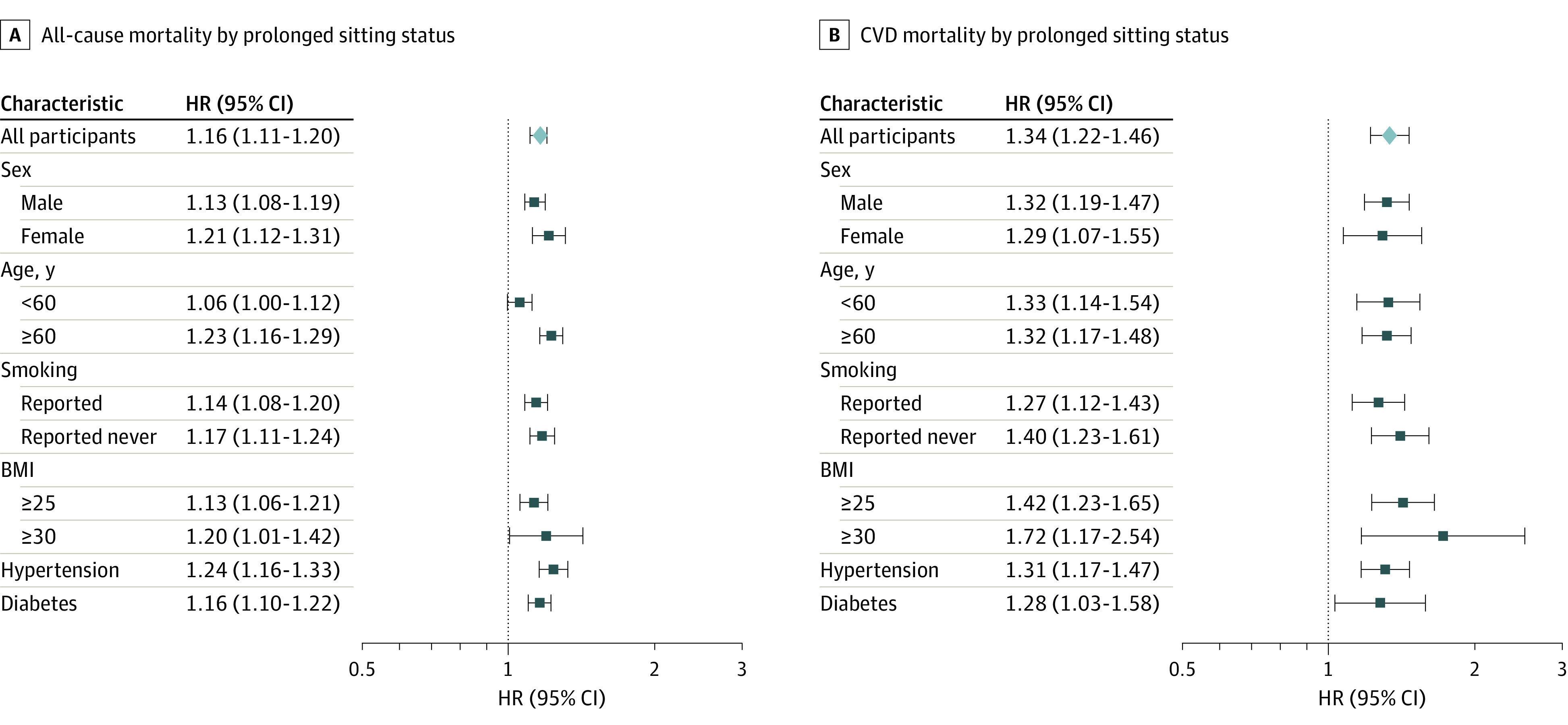
Adjusted Hazard Ratios (HRs) for Mortality by Prolonged Sitting Status Graphs show HRs for all-cause mortality (A) and cardiovascular disease (CVD) mortality (B), with nonsitters as the reference. All HRs are adjusted for sex, age, education, smoking, drinking, and body mass index (BMI; calculated as weight in kilograms divided by height in meters squared).

In all subgroup analyses, individuals mostly sitting at work exhibited a higher all-cause mortality risk, compared with their mostly nonsitting counterparts (Figure 1A). We observed significantly increased all-cause mortality among men (HR, 1.13; 95% CI, 1.08-1.19), women (HR, 1.21; 95% CI, 1.12-1.31), people younger than 60 years (HR, 1.06; 95% CI, 1.00-1.12), people aged 60 years and older (HR, 1.23; 95% CI, 1.16-1.29), smokers (HR, 1.14; 95% CI, 1.08-1.20), never smokers (HR, 1.17; 95% CI, 1.11-1.24), and people with chronic conditions, such as diabetes and hypertension. However, no significant differences were observed between the alternating sitting and nonsitting group and the mostly nonsitting group for all-cause mortality (Table 2).

A similar pattern was found for CVD mortality, because mostly sitting at work was associated with significantly higher risk among men (HR, 1.32; 95% CI, 1.19-1.47), women (HR, 1.29; 95% CI, 1.07-1.55), individuals younger than 60 years (HR, 1.33; 95% CI, 1.14-1.54), individuals aged 60 years or older (HR, 1.32; 95% CI, 1.17-1.48), smokers (HR, 1.27; 95% CI, 1.12-1.43), never smokers (HR, 1.40; 95% CI, 1.23-1.61), and, as before, among people with chronic conditions (Figure 1B). In sensitivity analyses using the expanded CVD criteria, which consisted of cardiovascular disease (*ICD-9* codes 390-459), plus diabetes (*ICD-9* code 250) plus kidney diseases (*ICD-9* codes 580-589) as a composite outcome, we obtained HRs for occupational prolonged sitting similar to the primary results (eTable 2 in Supplement 1). The results of the sensitivity analyses further confirm the primary findings.

**Figure 2.**
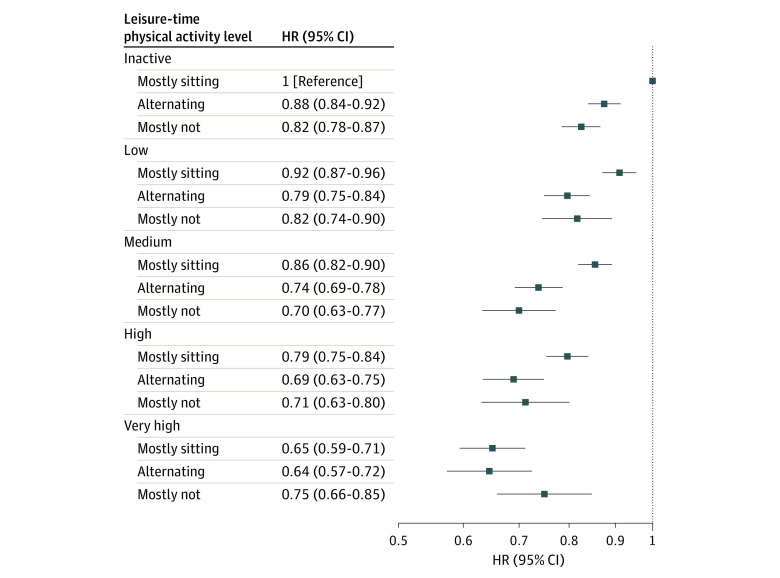
Adjusted Hazard Ratios (HRs) by Occupational Sitting Status Across 5 Leisure-Time Physical Activity Levels HRs are adjusted for sex, age, education, smoking, drinking, and body mass index. Leisure-time physical activity levels are classified as inactive (<3.75 metabolic equivalent of task [MET] hours per week or <5 minutes per day), low active (3.75-7.49 MET hours per week or 15 minutes per day), medium active (7.50-16.49 MET hours per week or 30 minutes per day), high active (16.50-25.49 MET hours per week or 60 minutes per day), and very high active (≥25.50 MET hours per week or ≥90 minutes per day).

As shown in Figure 2, with the inactive, mostly sitting group as the reference, we analyzed all-cause mortality risk across the 3 occupational sitting groups and the 5 levels of LTPA. At each LTPA level from inactive to high, individuals mostly sitting at work had significantly higher risks than those alternating sitting and nonsitting, and those mostly nonsitting, whereas the HRs estimated for these latter 2 groups were quite similar. At very high LTPA, no substantial differences were observed between mostly sitting (HR, 0.65; 95% CI, 0.59-0.71), alternating sitting and nonsitting (HR, 0.64; 95% CI, 0.57-0.72), and mostly nonsitting (HR, 0.75; 95% CI, 0.66-0.85) (Figure 2). Individuals mostly sitting at work engaging in low LTPA were less at risk than the reference group (HR, 0.92; 95% CI, 0.87-0.96), but more at risk than individuals alternating sitting and nonsitting engaged in the same LTPA level (HR, 0.79; 95% CI, 0.75-0.84) and more at risk than individuals mostly nonsitting engaged in no (HR, 0.83; 95% CI, 0.78-0.87) and low (HR, 0.82; 95% CI, 0.74-0.90) LTPA (Figure 2). Only at medium LTPA, the risk for individuals mostly sitting at work became similar (HR, 0.86; 95% CI, 0.82-0.90) to that of individuals alternating sitting and nonsitting and to that of individuals mostly nonsitting at no and low LTPA. At medium, high, and very high LTPA, the HRs estimated for the alternating sitting and nonsitting group were similar to those estimated for the mostly nonsitting group, ranging between 0.65 and 0.75. To reach the same risk, individuals mostly sitting at work needed to engage in higher level LTPA (Figure 2).

In Table 3, our analysis reveals that at PAI below 100, individuals mostly sitting at work had a higher adjusted HR for all-cause mortality than those mostly not sitting at work. Specifically, when using mostly sitting and physical inactivity as the reference, individuals mostly sitting at work with a PAI of 1 to 49 had an adjusted HR of 0.88 (95% CI, 0.85-0.91), and those with a PAI of 50 to 99 had an adjusted HR of 0.86 (95% CI, 0.82-0.90). These HRs were significantly higher than those for nonsitters at the same PAI levels (Table 3). Conversely, for individuals with a PAI of 100 to 149 and greater than 150, the HRs for mostly sitting and nonsitting at work were similar. For PAI of 100 to 149, the HR was 0.77 (95% CI, 0.72-0.82) for sitting vs 0.74 (95% CI, 0.64-0.85) for nonsitting. For PAI greater than 150, the HR was 0.70 (95% CI, 0.65-0.75) for sitting vs 0.69 (95% CI, 0.62-0.78) for nonsitting.

**Table 3.** Adjusted HRs for All-Cause Mortality by Occupational Sitting Status Across 5 PAI Levels

Occupational sitting status	Adjusted HR (95% CI)^a^				
	Inactive	PAI score 1-49	PAI score 50-99	PAI score 100-149	PAI score >150
Mostly sitting	1 [Reference]	0.88 (0.85-0.91)	0.86 (0.82-0.90)	0.77 (0.72-0.82)	0.70 (0.65-0.75)
Alternating sitting and nonsitting	0.87 (0.84-0.91)	0.78 (0.74-0.81)	0.78 (0.73-0.83)	0.68 (0.62-0.75)	0.67 (0.61-0.74)
Mostly nonsitting	0.83 (0.79-0.87)	0.77 (0.72-0.83)	0.74 (0.67-0.82)	0.74 (0.64-0.85)	0.69 (0.62-0.78)

Abbreviations: HR, hazard ratio; PAI, personal activity intelligence.

^a^ All HRs are adjusted for sex, age, education, smoking, drinking, and body mass index.

## Discussion

In this cohort study, we found that individuals who mostly sit at work had higher mortality risks than those who mostly do not sit at work. This finding held across numerous subpopulations. Our study finds that alternating between sitting and nonsitting at work or increasing LTPA can alleviate the harms. For example, individuals who alternated between sitting and nonsitting at work experienced a 16% reduction in all-cause mortality compared with individuals who mostly sat at work. Furthermore, we found that individuals who mostly sit at work but engage in high LTPA have all-cause mortality comparable to that of individuals who mostly do not sit at work but had lower LTPA.

Our findings suggest that the detrimental effects are more salient in individuals engaged with no to low level of physical activity, aligning with the 2018 update from the Physical Activity Guidelines Advisory Committee.^35^ Our observed HR of 1.16, comparing individuals mostly sitting at work with those mostly not sitting at work, aligns with a recent cohort study by Eklund et al,^20^ where an HR of 1.15 was reported for all-cause mortality when comparing 8 to 11 hours of prolonged sitting with less than 4 hours. Although our study differs in approach, it is consistent with the aforementioned meta-analysis.^20^ Overall, our findings from a large prospective cohort help to strengthen the increasingly accumulating evidence linking a sedentary lifestyle and health risks. As previously noted, evidence on health risks associated with occupational sitting is less evident because the heterogeneity of study designs and measures makes it challenging to draw definitive conclusions.^10,17^

Furthermore, we bolstered our findings by using the PAI metric. We convert the standard MET-hour per week into a PAI metric contingent more on intensity. This allows us to estimate the required level of intensity, as quantified by PAI, needed to mitigate the increased risk associated with prolonged occupational sitting. Notably, we observed that the elevated mortality risk is substantially attenuated among individuals with a PAI score exceeding 100. Our study contributes to the existing literature by demonstrating the applicability and advantages of PAI in the context of a large Asian population. PAI offers a user-friendly and comprehensive framework, making it easily understandable for a broad audience while promoting optimal health benefits.

Several explanations have been proposed to explain the harms of prolonged sitting. These include a lack of exercise of the large muscles in the lower limbs and trunk with increased blood flow to lower extremities, as well as the presence of a biomarker for low-grade inflammation.^36,37^ Such factors can lead to reduced insulin action,^38^ diabetes, obesity, metabolic syndrome,^39^ and reduced kidney function.^40,41^ Because individuals working long hours often share many characteristics with those who have a sedentary lifestyle, it is not surprising that a similar amplification of CVD risks has been reported in a number of studies.^15,21,22,42^

Our findings have several implications for interventions. First, the observed attenuated risk among individuals who alternate between sitting and nonsitting at work suggests that incorporating regular breaks in work settings can be beneficial. These breaks can be facilitated through the use of wearable devices^43^ or structured employer-designed break times. Experimental studies have explored the effectiveness of such interventions, revealing positive effects on metabolic outcomes.^44^ Practical solutions such as standing tables and activity-permissive workstations can also reduce sedentary time at work effectively without compromising work performance.^45^ Second, our findings offer reassurance that the increased risks can be offset by an extra 15 to 30 minutes per day of exercise per day or by participating in more physically intense activities. Employers can play a role in facilitating this by providing designated areas for LTPA or offering company-sponsored group activities.

### Strengths and Limitations

This study has a number of strengths. First, it is based on a large single cohort, ensuring consistent definitions of prolonged occupational sitting and physical activity across the entire cohort, which is in contrast to the heterogeneity often encountered in pooled studies.^19,20^ Second, the use of MET hours per week as a metric to quantify physical activity has been validated in a previous, highly cited publication.^27^ Third, study participants underwent comprehensive medical examinations, providing biological data for adjustment of potential confounding factors. Fourth, our ability to link the data to the national death registry and the exclusion of individuals with preexisting health conditions and those who died within the first 2 years of cohort entry minimizes the potential for reverse causation.

The present study has also several notable limitations. First, self-reporting of sitting and physical activity introduces possible bias, as participants may tend to provide socially desired responses, potentially overreporting their exercise levels and underreporting their sitting time compared with those measured by an objective method.^46^ However, the extensive nature of the questionnaire, comprising approximately 100 questions during check-ups, reduces the likelihood of substantial overreporting or underreporting. Second, the self-payment model for health check-ups may have attracted individuals with higher socioeconomic status, potentially impacting the generalizability of the findings. However, our risk calculations were based on internal comparison of subgroups, thus minimizing the effect of socioeconomic status. Third, although we categorized prolonged occupational sitting into 3 groups to demonstrate a dose-response effect, a precise quantification of the number of hours spent sitting at work every day was not available. Fourth, we have adjusted for education but not income because our income data have substantial missing values. Although highly correlated, some effects we have observed could be due to income differences beyond the educational level for which we adjusted. Fifth, during the extended follow-up period, the self-reported physical activity questionnaire underwent a few modifications, potentially leading to measurement incompatibility. However, the prevalence of medium and above LTPA engagement (equivalent to 150 minutes per week) remained relatively consistent, demonstrating good reliability between 1996 and 2017 among the cohort participants (eFigure 1 in Supplement 1).

## Conclusions

The serious risks associated with prolonged occupational sitting can be mitigated by incorporating regular breaks and engaging in additional physical activity. Systemic changes, such as more frequent breaks, standing desks, designated workplace areas for physical activity, and gym membership benefits, can help reduce risk.

## Notes

Supplement 1. **eFigure 1.** Data Flow Diagram Including Excluded and Missing Data **eFigure 2.** Percentage of Participants Physically Active and Percentage of Participants Mostly Sitting at Work in the MJ Cohort Between 1996 and 2017 **eTable 1.** Occupational Sitting Status Distribution at First and at Second Visit, MJ Cohort 1996-2017 **eTable 2.** Adjusted Hazard Ratios (HRs) for Expanded CVD Mortality by Occupational Sitting Status Across Subgroups

Supplement 2.
Data Sharing Statement
