# Error in Funding/Support Statement

**DOI:** 10.1001/jamanetworkopen.2024.2037

**Published:** 2024-02-14

In the Original Investigation titled “Occupational Sitting Time, Leisure Physical Activity, and All-Cause and Cardiovascular Disease Mortality,”^1^ published January 19, 2024, there was a missing grant number in the Funding/Support statement. This article has been corrected.^1^
