# Supplementary material for: Occupational Sitting Time, Leisure Physical Activity, and All-Cause and Cardiovascular Disease Mortality
Source: JAMA Netw Open. 2024 Jan 19;7(1):e2350680. doi: 10.1001/jamanetworkopen.2023.50680 (PMC10799265; doi:10.1001/jamanetworkopen.2023.50680)
Supplement: Supplement 1. — eFigure 1. Data Flow Diagram Including Excluded and Missing Data eFigure 2. Percentage of Participants Physically Active and Percentage of Participants Mostly Sitting at Work in the MJ Cohort Between 1996 and 2017 eTable 1. Occupational Sitting Status Distribution at First and at Second Visit, MJ Cohort 1996-2017 eTable 2. Adjusted Hazard Ratios (HRs) for Expanded CVD Mortality by Occupational Sitting Status Across Subgroups [file jamanetwopen-e2350680-s001.pdf]

Supplemental Online Content

Gao W, Sanna M, Chen YH, Tsai MK, Wen CP. Occupational sitting time, leisure physical activity, and all-cause and cardiovascular disease mortality. *JAMA Netw Open*. 2024;7(1):e2350680. doi:10.1001/jamanetworkopen.2023.50680

eFigure 1. Data Flow Diagram Including Excluded and Missing Data

eFigure 2. Percentage of Participants Physically Active and Percentage of Participants Mostly Sitting at Work in the MJ Cohort Between 1996 and 2017

eTable 1. Occupational Sitting Status Distribution at First and at Second Visit, MJ Cohort 1996-2017

eTable 2. Adjusted Hazard Ratios (HRs) for Expanded CVD Mortality by Occupational Sitting Status Across Subgroups

This supplemental material has been provided by the authors to give readers additional information about their work.

© 2024 Gao W et al. *JAMA Network Open.*

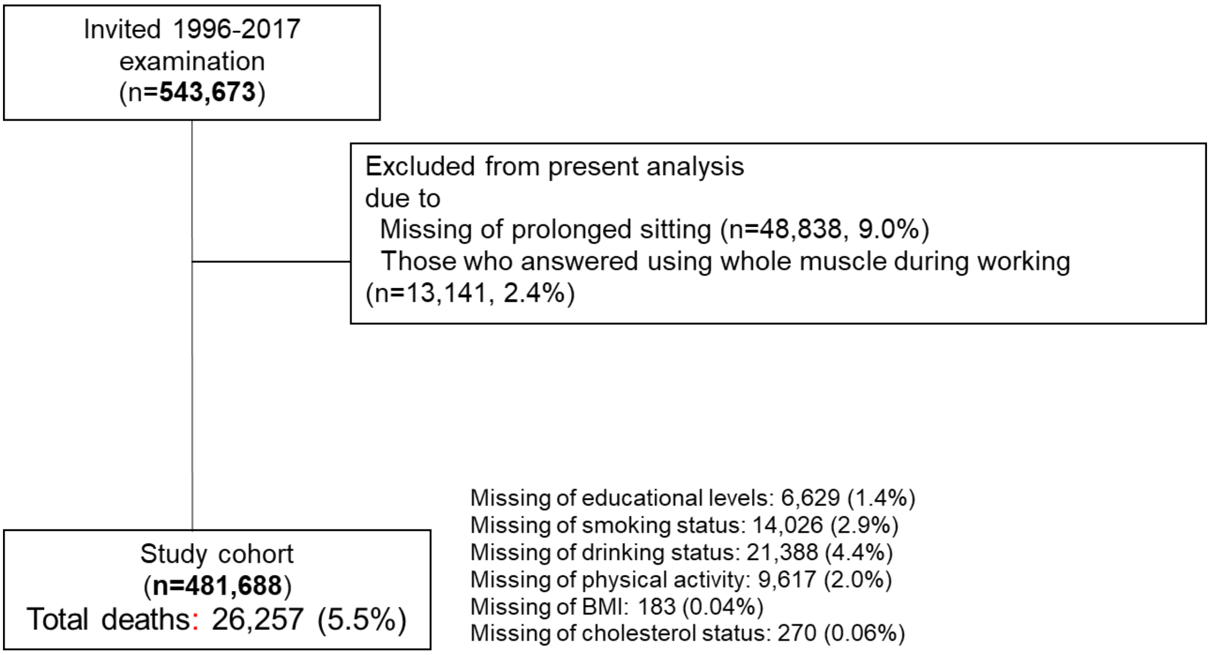

eFigure 1 Data Flow Diagram Including Excluded and Missing Data

© 2024 Gao W et al. *JAMA Network Open.*

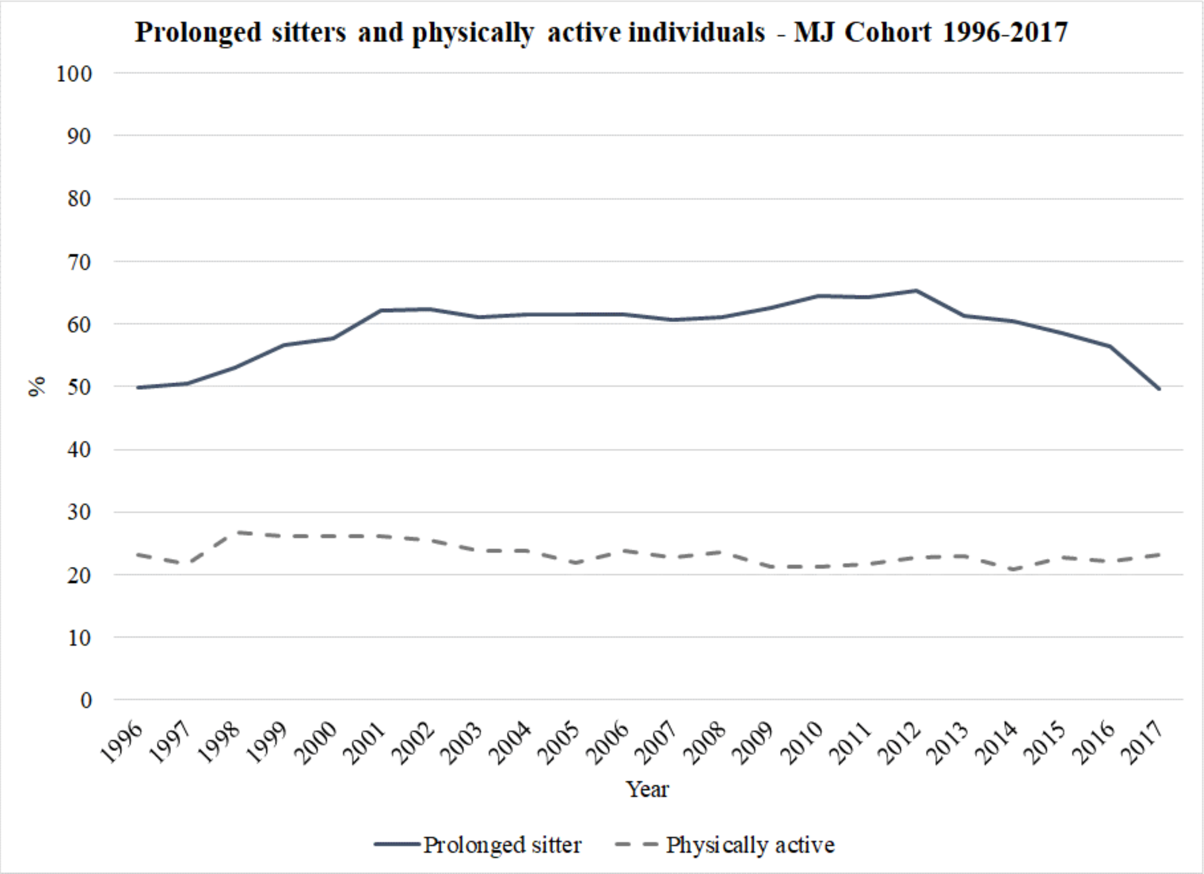

eFigure 2 Percentage of Participants Physically Active and Percentage of Participants Mostly Sitting at Work in the MJ Cohort Between 1996 and 2017

© 2024 Gao W et al. *JAMA Network Open.*

eTable 1 Occupational Sitting Status Distribution at First and at Second Visit, MJ Cohort 1996-2017

**Second visit \ First visit**	**Mostly sitting [N, (%)]**	**Alternating sitting and non-sitting [N, (%)]**	**Mostly non-sitting [N, (%)]**	**Total [N, (%)]**
**Mostly sitting [N, (%)]**	106,994 (53.9%)	18,439 (9.3%)	2,778 (1.4%)	128,211 (64.5%)
**Alternating sitting and non-sitting [N, (%)]**	14,708 (7.4%)	32,515 (16.4%)	6,101 (3.1%)	53,324 (26.8%)
**Mostly non-sitting [N, (%)]**	2,228 (1.1%)	5,632 (2.8%)	9,270 (4.7%)	17,130 (8.6%)
**Total [N, (%)]**	123,930 (62.4%)	56,586 (28.5%)	18,149 (9.1%)	198,665 (100%)

The Spearman’s rank correlation coefficient between occupational sitting status at the first visit and occupational sitting status at the second visit was equal to 0.61 (p<0.001).

© 2024 Gao W et al. *JAMA Network Open.*

eTable 2 Adjusted Hazard Ratios (HRs) for Expanded CVD Mortality by Occupational Sitting Status Across Subgroups

Population		Expanded CVD		
		**Deaths**	**HR***	**[95% CI]**
**All participants**	Mostly sitting	4,646	1.46	[1.35, 1.58]
	Alternating sitting and non-sitting	1,928	1.13	[1.04, 1.23]
	Mostly non-sitting	962	Ref.	
**Men**	Mostly sitting	2,426	1.42	[1.29, 1.55]
	Alternating sitting and non-sitting	946	1.14	[1.03, 1.26]
	Mostly non-sitting	726	Ref.	
**Women**	Mostly sitting	2,220	1.42	[1.22, 1.66]
	Alternating sitting and non-sitting	982	1.06	[0.91, 1.25]
	Mostly non-sitting	236	Ref.	
**Age<60**	Mostly sitting	1,288	1.44	[1.26, 1.63]
	Alternating sitting and non-sitting	787	1.19	[1.04, 1.35]
	Mostly non-sitting	409	Ref.	
**Age≥60**	Mostly sitting	3,358	1.47	[1.33, 1.63]
	Alternating sitting and non-sitting	1,141	1.09	[0.98, 1.22]
	Mostly non-sitting	553	Ref.	
**Smokers**	Mostly sitting	1,675	1.43	[1.28, 1.59]
	Alternating sitting and non-sitting	653	1.09	[0.97, 1.23]
	Mostly non-sitting	503	Ref.	
**Never-smokers**	Mostly sitting	2,722	1.47	[1.31, 1.65]
	Alternating sitting and non-sitting	1,165	1.15	[1.02, 1.30]
	Mostly non-sitting	391	Ref.	
**BMI≥25**	Mostly sitting	2,061	1.50	[1.32, 1.71]
	Alternating sitting and non-sitting	867	1.17	[1.02, 1.34]
	Mostly non-sitting	363	Ref.	
**BMI≥30**	Mostly sitting	376	1.47	[1.07, 2.00]
	Alternating sitting and non-sitting	147	1.08	[0.77, 1.51]
	Mostly non-sitting	57	Ref.	
**Non-hypertensives**	Mostly sitting	1,393	1.42	[1.25, 1.61]
	Alternating sitting and non-sitting	653	1.08	[0.94, 1.24]
	Mostly non-sitting	395	Ref.	
**Hypertensives**	Mostly sitting	3,253	1.42	[1.28, 1.57]

© 2024 Gao W et al. *JAMA Network Open.*

	Alternating sitting and non-sitting	1,275	1.08	[0.97, 1.20]
	Mostly non-sitting	567	Ref.	
**Non-diabetics**	Mostly sitting	2,653	1.32	[1.21, 1.45]
	Alternating sitting and non-sitting	1,389	1.11	[1.00, 1.22]
	Mostly non-sitting	729	Ref.	
**Diabetics**	Mostly sitting	1,693	1.47	[1.27, 1.71]
	Alternating sitting and non-sitting	539	1.04	[0.88, 1.23]
	Mostly non-sitting	233	Ref.	

*All HRs are adjusted for gender, age, education, smoking, drinking, and body mass index (BMI).

© 2024 Gao W et al. *JAMA Network Open.*
