# Supplementary material for: Occupational Sitting Time, Leisure Physical Activity, and All-Cause and Cardiovascular Disease Mortality
Source: JAMA Netw Open. 2024 Jan 19;7(1):e2350680. doi: 10.1001/jamanetworkopen.2023.50680 (PMC10799265; doi:10.1001/jamanetworkopen.2023.50680)
Supplement: Supplement 2. — Data Sharing Statement [file jamanetwopen-e2350680-s002.pdf]

Data Sharing Statement

Gao. Occupational Sitting Time, Leisure Physical Activity, and All-Cause and Cardiovascular Disease Mortality. *JAMA Netw Open*. Published January 19, 2024.
doi:10.1001/jamanetworkopen.2023.50680

Data
Data available: Yes
Data types: Deidentified participant data
How to access data: The Taiwan MJ Cohort is available to the worldwide research community and offers opportunities for collaboration. Those applying for data access should contact the MJ Health Research Foundation at http://www.mjhrf.org/
When available: With publication

Supporting Documents
Document types: None

Additional Information
Who can access the data: researchers whose proposed use of the data has been approved
Types of analyses: for any purpose
Mechanisms of data availability: after approval of a proposal
